# Regional Adiposity, Cognition and Brain Volumes in Older Adults with Type 2 Diabetes: Sex‐Specific Associations

**DOI:** 10.1002/alz70860_104389

**Published:** 2025-12-23

**Authors:** Ethel Boccara, Sapir Golan, Ithamar Ganmore, Ramit Ravona‐Springer, Mery Ben‐Meir, Abigail Livny, Yael Inbar, Yossi Azuri, Aron Weller, Michal S Beeri

**Affiliations:** ^1^ The Joseph Sagol Neuroscience Center, Sheba Medical Center, Tel Hashomer, Israel; ^2^ Department of Psychology, Bar Ilan University, Ramat Gan, Israel; ^3^ Sackler Faculty of Medicine, Tel Aviv University, Tel Aviv, Israel; ^4^ Department of Diagnostic Imaging, Sheba Medical Center, Tel Hashomer, Israel; ^5^ Maccabi Health Services, Tel Aviv, Israel; ^6^ Herbert and Jacqueline Krieger Klein Alzheimer's Research Center at Rutgers Brain Health Institute, New Brunswick, NJ, USA

## Abstract

**Background:**

Adiposity and Type 2 diabetes (T2D) are linked to cognitive decline and dementia, but findings on global adiposity, typically measured by body mass index (BMI), remain inconsistent, especially in older adults. Given sex differences in fat distribution, regional adiposity may offer critical insights into these associations. This study examined whether regional fat deposition relates to cognitive function and Alzheimer's disease (AD)‐related brain volumes in older adults with T2D from the Israel Diabetes and Cognitive Decline (IDCD) study.

**Methods:**

The analysis included 118 participants (48.3% women) with MRI‐based abdominal fat measurements, of whom 96 underwent neuropsychological assessments and 57 had brain MRI scans. Regional adiposity measures included hepatic fat, pancreatic fat, visceral adipose tissue (VAT), and subcutaneous adipose tissue (SAT). Cognitive outcomes encompassed global cognition, episodic memory, working memory, executive function, and language. Brain volumes assessed included total grey matter (GM), hippocampus, and prefrontal regions (inferior, middle, and superior frontal gyri: IFG, MFG, and SFG). Linear regression models adjusted for age, sex, education, BMI, and intracranial volume.

**Results:**

No associations were found between hepatic fat, pancreatic fat, VAT%, or SAT% and cognitive performance in either sex. However, sex‐specific differences emerged in brain volume associations. In women, higher hepatic fat % was associated with greater IFG volume (β=0.51, *p* = 0.04), while no such relationship was found in men (β=0.04, *p* = 0.82; p for interaction = 0.73). Hepatic fat % was not linked to other AD‐related brain regions. In men, higher pancreatic fat % correlated with smaller SFG volume (β=‐0.44, *p* = 0.03), whereas no such association was found in women (β=0.43, *p* = 0.23; p for interaction = 0.40). A significant interaction was observed between pancreatic fat % and GM volume (*p* for interaction = 0.04). Across the whole sample, higher VAT% was associated with smaller MTG volume (β=‐0.31, *p* = 0.03), while higher SAT% correlated with larger MTG volume (β=0.36, *p* = 0.02). VAT% and SAT% were not linked to GM, SFG, MFG, or IFG.

**Conclusion:**

Regional adiposity is differentially associated with brain structure in older adults with T2D, with notable sex‐specific patterns. These findings underscore the need to consider fat distribution and sex differences when investigating the impact of adiposity on cognition and brain health in aging populations.

